# Supplementary material for: Computational simulations and Ca2+ imaging reveal that slow synaptic depolarizations (slow EPSPs) inhibit fast EPSP evoked action potentials for most of their time course in enteric neurons
Source: PLoS Comput Biol. 2022 Jun 13;18(6):e1009717. doi: 10.1371/journal.pcbi.1009717 (PMC9232139; doi:10.1371/journal.pcbi.1009717)
Supplement: Attachment — Submitted filename: Review.docx [file pcbi.1009717.s001.docx]

# General

This is an important paper that helps to elucidate some aspects of synaptic processing in the phylogenetically 1^st^ nervous system to evolve before the central nervous system in animals such as hydra and hemichordates. The enteric nervous system is enormously complex and its importance is emphasised by its necessity to life, as well as by being a major source of sensory input to the central nervous system via the afferent vagus nerve.

The authors used the neuron simulation environment and a modified Hodgkin-Huxley formalism to model the synaptic activity of slow depolarising synaptic input to myenteric colonic S cell. A major computational prediction was that the slow depolarising potentials would have a biphasic postsynaptic effect. With the initial phase of the depolarising potentials having an excitatory effect evoking action potentials in the postsynaptic S cell while the latest sustained component was predicted to fail to evoked action potentials. This biphasic effect was also confirmed when slow depolarising potentials were paired with sub-or supra-threshold fast postsynaptic potentials. GABAa (fast kinetics) and GABAc (slow kinetics) mediated postsynaptic depolarising potentials were also modelled. Activation of GABAc potentials were predicted to initially enhance but later inhibit sub- or supra-threshold GABAa evoked potentials when these were applied at various times during the evolution of the GABAa depolarising potential.

The authors used Ca2+ imaging as a surrogate for neuronal activation to demonstrate that slow synaptic depolarisations can indeed suppress fast depolarising potentials for some neurons in an experimental setting.

I recommend that this study be published subject to the major comments being addressed in the discussion.

# Major Comments

1. There are many sources of membrane noise in individual neurons and the synaptic network. These derived, inter-alia, from thermal sources, stochastic fluctuations between open and close states of ion channels and synaptic transmission. It seems that the modelling did not include a variable for such noise. Could the authors comment on how this might affect the action potential firing probability of neurons within the model.

2. The enteric nervous system is spontaneously active physiologically receiving input from the luminal epithelium and smooth muscle contractions. Could the authors comment on this and suggest how this might alter their model and its ability to simulate a physiologically relevant environment.

3. Could immunohistochemistry have been performed to identify S cell subtypes?

# Minor Comments

The manuscript had few typographical errors.

Line 65:… using live Ca2+ rather than Ca.

In line 88: provide reference to research paper.

In-line 204: Modelled GABAa mediated responses…

In-line 207: In contrast the modelled GABAc…

In-line 295: We tested whether small slow synaptic depolarisations could be inhibitory… I have found this confusing because the figure 5 legend describes large synaptic depolarisations rather than small as described here.
