# Supplementary material for: Computational simulations and Ca2+ imaging reveal that slow synaptic depolarizations (slow EPSPs) inhibit fast EPSP evoked action potentials for most of their time course in enteric neurons
Source: PLoS Comput Biol. 2022 Jun 13;18(6):e1009717. doi: 10.1371/journal.pcbi.1009717 (PMC9232139; doi:10.1371/journal.pcbi.1009717)
Supplement: Attachment — Submitted filename: Response to reviewers JCB.docx [file pcbi.1009717.s002.docx]

We thank the reviewers for their thoughtful suggestions most of which we have incorporated in the revised manuscript with the revised material being in red in the resubmitted text. Reviewers’ comments are in italics below, our responses are in plain text.

**Reviewer 1:**

*There are many sources of membrane noise in individual neurons and the synaptic network. These derived, inter-alia, from thermal sources, stochastic fluctuations between open and close states of ion channels and synaptic transmission. It seems that the modelling did not include a variable for such noise. Could the authors comment on how this might affect the action potential firing probability of neurons within the model.*

All these elements of the noise are interesting to consider. Some will affect the strength of the connections, which translates to the amplitudes of the fast EPSPs in particular with electrophysiological experiments indicating that these amplitudes can vary by several millivolts between successive action potentials. This variability dominates all other random sources of variation, although transmission through polysynaptic pathways can lead to significant variability in latency of fast EPSPs evoked via such pathways, which in turn affects summation and the overall size and duration of the compound fast EPSPs evoked by an electrical stimulus. Both these issues would be expected to play major roles in a model of a realistic circuit and the variability introduced would be increased somewhat by the other sources of variability mentioned by the reviewer. We have added a short discussion of this physiological variability to the discussion of the physiological significance of our data (lines 553-565 of manuscript). Nevertheless, our model study was designed to remove all such variability by assigning a single input neuron to represent all neurons producing a particular type of synaptic input to the test neuron. This allowed the very specific question as to whether slow EPSPs can enhance the probability of firing of a neuron in response to fast EPSP inputs without having to consider other sources of variability, including differences in conduction delays, etc.

The various contributions of random variations, both static (eg connectivity) and dynamic (transmitter release probability, etc), can be explored in future works or by interested readers using the provided codes which incorporate provisions to include such parameters.

*2. The enteric nervous system is spontaneously active physiologically receiving input from the luminal epithelium and smooth muscle contractions. Could the authors comment on this and suggest how this might alter their model and its ability to simulate a physiologically relevant environment.*

We agree that this is an important experiment to undertake with the model when it is configured to represent a more realistic network incorporating all the different functional elements of the circuit. However, it is a much larger project than this present one and requiring both a major increase in circuit size and very extensive trials. It represents one of our ultimate goals. We have included a very brief comment in the Discussion to this effect (manuscript line 559-560).

*3. Could immunohistochemistry have been performed to identify S cell subtypes?*

In principle, this could be incorporated into our experiments as *post hoc* immunohistochemistry on preparations used for Ca imaging is an important tool in many of our other studies. It would extend this present study in some potentially interesting directions, especially in possibly discriminating between the neurochemical subtypes of neurons that increased firing, those that decreased firing and those whose firing was unchanged. Unfortunately, identifying ganglia from which recordings are made is a very difficult task in the proximal colon and we were unable to obtain satisfactory staining with several of the preparations examined. This reduced the potential sample size to the point where meaningful comparisons could not be made and as a result we have chosen not to present the limited data that we obtained.

*The manuscript had few typographical errors.*

*Line 65:… using live Ca2+ rather than Ca.*

Corrected

*In line 88: provide reference to research paper.*

This is a general comment covering 30+ years of electrophysiological and morphological studies both published and unpublished. This work is best summarised in the historical review already cited.

*In-line 204: Modelled GABAa mediated responses…*

Corrected

*In-line 207: In contrast the modelled GABAc…*

Corrected

*In-line 295: We tested whether small slow synaptic depolarisations could be inhibitory… I have found this confusing because the figure 5 legend describes large synaptic depolarisations rather than small as described here.*

We thank the reviewer for pointing this discrepancy out and have added a new Figure 5 and Figure description to clarify this point.

**Reviewer 2:**

*1) In Figure 1, the authors argue that their model replicates physiological recordings and is therefore valid. While this appears reasonable, it is less clear how the authors chose parameters for all the conductances and their kinetics. For example, the peak Na+ and delayed rectifier conductances (Table 1) are said to have values of 0.01, but with no units! The Kv7.2 conductance as value of 0.15 (no units). Generally, the magnitude of m-conductance is much smaller than that of voltage-gated Na+ conductance and the delayed rectifier K+ conductance. The table should be carefully edited and the strategy for tuning the model by setting parameters should be explained more clearly. It would also help to add a citation for evidence that the reversal potential for chloride is -35mV. Also, what was the resting potential, input resistance and capacitance of the model? – these often are important in tuning a model.*

The values for the maximum conductance of the channels are just coefficients and do not have units. The fourth column in Table 1, indicates the references from which these parameters are derived and we have added a statement (lines 751-752) to clarify this. We have added reference to Kang et al 2003 as suggested by reviewer 3 to support the use of a reversal potential for Cl^-^ of -35mV. The resting membrane potential and input resistance was set by the model which runs using the NEURON simulation environment (we have added the basic model cell parameters including membrane capacitance to the beginning of the Materials and Methods, pages 577-582) in the first few milliseconds at the start of each run using the set reversal potentials and resting conductances. This is best shown for membrane potential in Fig 1J.

*2) In many of the figures, starting with fig 1, the lettering is way too small. Figure 1 would also be improved by denoting physiological recordings and simulations in the figure, not simply in the legend. It took a while to decode this and annotate the manuscript, which detracts from the story.*

We have modified all Figures to incorporate larger lettering and other changes suggested by reviewer 2 and reviewer 3. For Figure 1 we have noted in each panel whether the records shown are from physiological recordings or simulations in the figure itself.

*3) The slow EPSP is modeled based on the kinetics of cyclic AMP generation and downstream kinases. While this may have been reasonable when the model was originally developed many years ago, it is now clear the Kv7 suppression is mediated by the hydrolysis of PIP2. Perhaps it is sufficient to use a model that simply replicates the kinetics and stimulus dependence of slow EPSPs (analogous to how fast PSPs were modeled).*

Both the conductance changes and the second messenger systems that mediate slow EPSPs in enteric neurons remain uncertain with several different GPCRs and target conductances involved for any one neuron. These probably include both the cAMP pathway we have modelled and the PIP_2_ hydrolysis pathway identified by the reviewer. The model of the slow EPSP simulates the modulation of a non-specified K channel. We chose it because while the GPCR/intracellular message system may differ from some physiological systems, the key question (as postulated by the reviewer) is how depolarization coupled with a reduced K^+^ conductance affects action potential generation which is independent of the mechanism that alters the K^+^ conductance. We have added a section in the text addressing this point (lines 467-471).

*4) Does Na+ channel inactivation play a critical role in the inhibition seen during slow EPSPs? – see line 437 in discussion. This seems highly likely. The authors could address the question in 3 ways. One would be to monitor the state if inactivation during the computational simulation. The second would be to inject a hyperpolarizing current during the slow EPSP to prevent the depolarization. Third, they could map the time course for the ‘inhibition’ during the slow epsp by probing it with fast PSPs and different time points and determining whether inhibition correlates with the degree of depolarization or the duration of depolarization.*

These are each interesting suggestions and it seems likely that Na^+^ channel inactivation would have some role in the inhibitory component of slow EPSPs, as highlighted in our Discussion. Nevertheless, our finding that in the absence of a slow delayed rectifier channel (Kv7.2 in these simulations) modelled neurons fire continuously without any decrement in frequency throughout a 500 ms imposed depolarization indicates that Na^+^ channel inactivation has a secondary role, if any, to the delayed rectifier. We have added this conclusion to the text (lines 456-459).

*5) Statistics – the methods (lines 766 to 770) describe statistical tests using unpaired t-test. This ready may have overlooked it, but did not see any reports of averaged data, variance or statistical tests. Given this approach, it would be more appropriate to describe the experimental results as exploratory findings that possibly demonstrate a proof of principle for further work.*

We thank the reviewer for pointing out this issue. We used the unpaired t-tests to compare the amplitudes of the Ca^2=^ transients that we studied and found that there were no significant differences between the amplitudes of control responses and those of neurons that responded after a conditioning stimulus train. But because more than half of the initially responsive neurons failed to respond after the conditioning train and the relationship between the amplitudes of Ca transients and neuron excitability is not necessarily linear, we chose not to include these data in the body of the Results. We have now added these findings to the end of the Materials and Methods (lines 808-815) to justify our focus on numbers of responding neurons. We have also added a sentence (lines 809-810) to indicate that the recovery of excitability in some neurons was taken to indicate that the inhibition of activity was indeed due to the conditioning stimulus train. We have altered the Results section accordingly (lines 352-353).

**Reviewer 3:**

*It would be informative to also show the results in the output neuron of the different stimulations on an expanded timescale (e.g. in an inset), such that amplitude changes and possibly small delays can be appreciated.*

Insets showing expanded tracts have been added to each of the relevant figures.

*Figure 2: it is not clear why the panels are organized like that. To me it seems more logic to show the synchronous and a-synchronous stimulation panels respectively above one another.*

The figure has been modified in the new version.

*The Ca imaging data are intriguing and important to provide support for the inhibitory role that an excitatory potential might have. However, the fact that some neurons start responding rather than being inhibited is a little bit detached from the rest of the paper, despite the fact that this is fairly well addressed in the discussion, with some options clearly explained as to why this might happen. I suggest to add a little bit of that explanation already in the results, to avoid that this section ends a bit on a confusing note, and only gets explained a couple of pages later.*

We thank the reviewer for this suggestion and have added a sentence containing some suggestions about the underlying mechanism for the excitation to the relevant part of the Results (lines 358-361).

*The authors refer to the guinea-pig study of Cherubini and North to suggest that the reversal potential of Cl in colonic neurons is around -35 mV. Interestingly this was also shown in mouse colonic neurons in a patch clamp study by Kang et al. DOI: 10.1152/ajpcell.00437.2002. There it was suggested that these neurons accumulate Cl. Could this be another possible explanation for the differences that the authors observe in their Ca imaging results? What if not all neurons accumulate Cl to the same extent? Could this explain why some and not other neurons are inhibited during the prolonged duration of the sEPSP?*

We thank the reviewer for drawing our attention to Kang et al 2002, which had slipped through our net. Wouters et al 2006 have reported that some myenteric neurons express NKCC1, which acts as a transporter to increase internal Cl^-^, but there is no evidence as to how many or which ones. We have assumed that all myenteric neurons have E_Cl_ at -35mV, but more data are needed to confirm this. Nevertheless, it is difficult to see how differences in Cl^-^_I_ would alter responsiveness unless there are changes in chloride ion conductance evoked by the 20 pulse stimulus trains, for which there is some evidence. This will need to be addressed in future biophysical analyses with neurochemical identification of relevant neurons. It is an interesting possibility, but too complex for this present study.

*I suggest to present the Ca imaging data a little bit differently. Wouldn’t it be more logic to show the number of responses per ganglion rather than only mentioning total of un/responding neurons summed over all the experiments?*

We believe that it makes little difference to express the data as total number of neurons rather than neurons per ganglion, because we are looking at intrinsically paired neurons, ie each neuron serves as its own control and we are only asking if it does or does not respond. Thus, responsive neurons per ganglion would allow us to calculate means and variances per ganglion for each condition, but as there is no reason to believe that there would be systematic differences between ganglia, per ganglion comparisons probably would provide no extra information.

*Furthermore, it is also important to show that the neurons were back to baseline before the 1P stimulus was given. Telling from the snapshots shown that is indeed the case, thus showing the values would strengthen the conclusions. Imagine that a Ca transient induced by the 20Hz stimulation lasts longer than 10 s, then the indicator would be still saturated and therefore a 1P stimulation would not be able to elicit a transient, not because there is no additional depolarization or AP but simply because the reporter is not available. As said, this does not seem to be the case as seen in the 2 images. However, the resting values (preferably not normalized) before the 20Hz and 1P stimulation would therefore be informative.*

We have added a short section about the time courses of the synaptic events and the Ca transients indicating that the interval between 20 pulse stimuli is more than sufficient for the Ca signal and the membrane potential to return to baseline (343-346). Unfortunately, we cannot rule out that some of the intracellular Ca response to a 20 pulse stimulus persists for longer than 10 s. This would be a physiological event. Nevertheless, it is highly unlikely that any failures of a neuron to respond to a single pulse stimulus would be due to saturation of the indicator as GCaMP6f has very fast kinetics and our other studies show can detect very much larger transients than are evoked by a single stimulus. Indeed, we have used the same protocol with closely spaced 20 pulse responses and seen no problem and in this study found that the amplitudes of the responses in neurons that responded to both test and control stimuli were identical.

*When referring to the role of nACh receptors in synaptic responses in the mouse ENS, it seems appropriate to also refer to the paper of Foong et al. 2015 (doi: 10.1523/JNEUROSCI.4175-14.2015).*

This citation has been added to the text and bibliography as suggested by the reviewer.

*Why is there only one temperature sensitive component in the model? It seems that only Nav1.3 is temperature sensitive? Is that correct? What effect did temperature have on the model? And does that match the Ca imaging results, which were recorded at RT?*

The effective temperature at which the other channels are functioning is incorporated as 36 degrees of Celsius in the equations. We did not explore the effect of temperature on the channels, because the electrophysiology was performed at 37^o^C and it is routine in the Ca imaging literature to compare room temperature Ca imaging with body temperature electrophysiology. Exploring the temperature sensitivity of the channels was not the focus of this study and may be explored in future works. Ion channels have low Q10s and so excitability comparisons like this are robust.

*The legend of Fig. 3 claims that the colors of the neurons are matched, but – at least in the pdf version of the manuscript- there is quite a bit of difference between the pinkish colors. Please correct.*

This has been modified in the new version.

*Introduction line 155: mv should be mV*

Corrected

*Legend of Fig.4: … are activated in the… I think a word is missing here.*

Corrected

*Line 391: although commonly used in the ENS research field, “AH” has not been defined in the current paper yet.*

We have added a definition of AH and relevant references (lines 407-409)

*Line 411: I assume the verb is missing: … which are expressed*

Corrected
